# Pathogenic built environment? Reflections on modeling spatial determinants of health in urban settings considering the example of COVID-19 studies

**DOI:** 10.3389/fpubh.2025.1502897

**Published:** 2025-03-17

**Authors:** Tobia Lakes, Tillman Schmitz, Henning Füller

**Affiliations:** ^1^Department of Geography, Faculty of Mathematics and Natural Sciences, Humboldt-Universität zu Berlin, Berlin, Germany; ^2^Integrative Research Institute on Transformations of Human Environment Systems (IRI THESys), Berlin, Germany

**Keywords:** spatial epidemiology, critical GIS, critical geography, urban health, density, human geography

## Abstract

The triad of host, agent, and environment has become a widely accepted framework for understanding infectious diseases and human health. While modern medicine has traditionally focused on the individual, there is a renewed interest in the role of the environment. Recent studies have shifted from an early-twentieth-century emphasis on individual factors to a broader consideration of contextual factors, including environmental, climatic, and social settings as spatial determinants of health. This shifted focus has been particularly relevant in the context of the COVID-19 pandemic, where the built environment in urban settings is increasingly recognized as a crucial factor influencing disease transmission. However, operationalizing the complexity of associations between the built environment and health for empirical analyses presents significant challenges. This study aims to identify key caveats in the operationalization of spatial determinants of health for empirical analysis and proposes guiding principles for future research. We focus on how the built environment in urban settings was studied in recent literature on COVID-19. Based on a set of criteria, we analyze 23 studies and identify explicit and implicit assumptions regarding the health-related dimensions of the built environment. Our findings highlight the complexities and potential pitfalls, referred to as the ‘spatial trap,' in the current approaches to spatial epidemiology concerning COVID-19. We conclude with recommendations and guiding questions for future studies to avoid falsely attributing a built environment impact on health outcomes and to clarify explicit and implicit assumptions regarding the health-related dimensions.

## 1 Introduction

The COVID-19 pandemic has brought public health to the forefront of research agendas worldwide. Beyond the immediate epidemiological characteristics of the novel coronavirus, spatial factors have been crucial in understanding the pandemic's dynamics, particularly regarding transmission pathways (e.g. the diffusion process or the influence of the built environment) (1, 2) and the effectiveness of public health interventions such as border closures, lockdowns, and other containment measures (3–5). A key focus has been on the role of urban environments, following a long-standing debate on detrimental health effects in urban settings, the so called “urban health penalty” (6). This concept suggests that urban settings can be detrimental to health by exposing individuals to various unhealthy settings (7). Early media reports during the pandemic highlighted these concerns, as did subsequent academic studies (8).

Since the onset of the COVID-19 pandemic, numerous studies have examined the role of the built environment as a spatial determinant of the disease's spread, seeking to understand its spatial nature and develop effective control strategies (9, 10, 86). This approach aligns with a broader trend in medicine and public health that emphasizes disease ecology—considering the environment as a critical factor in disease transmission (11). It marks a shift from the previous century's focus on host-pathogen interactions toward a more holistic view that includes environmental and social determinants of health (12). The related fields of spatial epidemiology (13), health geography (14, 15), and One Health (16, 17) have increasingly recognized the significance of contextual factors, including environmental conditions to social settings, as determinants of health in modeling health outcomes. Specific strains of this research concentrate on urban areas, considering them as complex systems comprising the physical and social environment and access to health and social services (18). Within this context, we adapt the definition of the built environment by Kaklauskas and Gudauskas (19) and consider built environment as the human-made surroundings that provide the setting for human activities, including buildings, parks or green space and supporting infrastructure. The built environment and its health relevance have gained significant attention in the last years (20, 21).

However, empirically modeling the impact of these spatial determinants on health outcomes, including COVID-19, remains a challenging task due to conceptual, methodological and data-related complexities. Issues such as the adequate choice of method (including categorization, data availability, unit of analysis and data quality) and the relationship between health and the built environment can serve as hidden entrance points for biased assumptions. These biases may influence understandings of underlying spatial processes and distribution patterns, as well as the positionalities of researchers. Singling out certain aspects such as spatial features and their type and importance of influence is methodologically and methodically difficult. A tendency of “invoking a vaguely-defined but often infinitely complex ‘environment' to ‘explain' variation in disease rates” has been criticized for a long time in this regard (22, 23). Spatial qualities of the built environment for example, such as (urban) density, built form or number of sites with a high frequency of interaction are frequently accessed through proxy variables in health modeling. These sources of inherent uncertainty are aggravated when different spatial scales are considered or when different spatial contexts are compared (24). Finally, many analyses of spatial determinants on health are limited by the general simplification that the study population is locally fixed at one point in space, such as the site of work or the site of residence. This overlooks the dynamic nature of human mobility, failing to account for how people move and interact across different spaces throughout their daily life. COVID-19, declared a “public health emergency of international concern” by the WHO on January 30, 2020, serves as a critical case for examining how the association between the built environment and health is reflected in research studies. Health is understood as “a state of complete physical, mental and social wellbeing and not merely the absence of disease or infirmity” (WHO definition) (25). The virus, primarily transmitted through airborne particles, finds optimal conditions for spread in settings where people come into close contact, particularly in indoor spaces like workplaces, schools, and public transport, i.e. most likely in densely populated urban environments (26, 27, 89). Hence, it seems reasonable that the built environment has been identified as a crucial component to consider in the management of the COVID-19 pandemic by researchers (2). We focus on the built environment in urban settings in this article because, despite its high importance in the context of COVID-19, there has been little attention given to the methodical and methodological challenges of addressing it in empirical studies.

Several health concepts have systematically addressed the built environment's influence on health, identifying both direct and indirect pathways through which human-made spaces can affect health (28–32). Some studies have reviewed and discussed the existing indicator approaches that capture the urban health system (29, 33). Direct and indirect pathways of how human-made spaces can harm or promote health are characterized depending on certain characteristics, e.g. availability, access or quality, and the local context (34). Nevertheless, the influence of the built environment is just one of many factors contributing to health outcomes, especially in urban neighborhoods. We are aware that several other factors also play an important role as determinants of health and COVID-19, particularly in urban neighborhoods (35).

Studies have hypothesized and analyzed multiple associations between the built environment and COVID-19 outcomes, focusing mainly on urban density and land use types. While land use types are mostly well-defined via their functions and the respective use, the term density, however, is a multifaceted concept with distinct meanings across various disciplines. In urban planning and architecture, density mainly refers to the concentration of people or structures in an area, often measured by population or building density. It is typically associated with benefits like efficient land use, reduced car dependency or increased social interaction (36, 37). In sociology, density (also called social density) may be defined as the number of interactions between people per unit of time (38). In contrast, epidemiological and public health studies often use density in the context of population distribution, particularly as it relates to health outcomes. With regard to the COVID-19 pandemic, concepts used to operationalize health effects of the built environment are often vague or overdetermined. As an example, Colin McFarlane differentiates four different types of relations often mixed together when the concept of “density” is evoked in COVID-19 related research. This eventually is also mixing different assumptions regarding health effects and transmission: “density as numbers of people *living in an urban area*, often a neighborhood, district, ward, or county; density as numbers *living in a house* [sometimes referred to as ‘overcrowding'—for a critique of the normative bias of this term see (38)]; density as numbers *gathering at sites*, including city centers, urban beaches and parks, shops, bars, cafes and restaurants, and so forth; and density as numbers *moving through space*, including transport systems, streets and the in-between spaces of city-center shopping, and so on” [(39), p. 1550 emph. in original].

Furthermore, the underlying hypothesized processes and directions that are to be captured by the different aspects of density are manifold in existing studies. On the one side, dense urban environments (including population, social activities, housing, and transport) are assumed to be associated with more interactions and greater proximity among people (40) or crowded housing conditions (41, 42). Both are associated with increased transmission and higher infection risks. On the other hand, dense areas may also be better environments in enforcing strict measures and policies as social distancing, in addition to having better access to health care facilities (43). Similarly contrasting are the hypotheses and findings of studies in terms of the associations with the diversity of land use. Urban neighborhoods with a mixed land use can on the one side encourage more gatherings and interactions, potentially leading to a rapid spread of COVID-19 (40). On the other side, mixed land use is associated with a lower need for long travel and may lead to a lower rate of mobility (44). Greenspace may improve immunity to COVID-19 through physical activity but also may promote close contacts and increase the risk of infections (41). Finally, characteristics of the built environment are frequently associated with other socio-economic, biophysical characteristics (45, 46) so that these should be accounted for to avoid misleading interpretation or attribution to the built environment only. In summary, concepts detailing the built environment are often vague. Several, and partly contrasting, associations between the built environment and COVID-19 are hypothesized, and the empirical findings vary between the different study areas, the spatial scale of analysis and the selected pandemic phases (35).

In recent years, constructivist and relational perspectives allowed new understandings also about the nexus of health and space (47). Research in geography, geoinformation science, social epidemiology, and other disciplines increasingly emphasizes the relevance of sociocultural aspects in understanding place, space and health (48). The traditional employment of the spatial as an unproblematized activity container does not suffice, given the relevance of constructed meaning and experiential aspects of place. Such a “health geography” approach highlights the contingencies of space. Space provides a context for social processes and interactions, but those processes and interactions also dynamically shape spaces and places (49). A recent study therefore calls for rethinking the underlying paradigms on urban health and the built environment and identifies the following four dimensions: conceptual, theoretical, methodological, and instrumental (50). Conceptual and theoretical questions of how to describe and capture the system under study have been extensively discussed. For example, identifying spatial risk factors within an assumed complex bundle of causes, in other words ‘multiple causation' health outcomes, has been criticized for a long time (22). Particularly with the increasing availability of data-driven approaches, modeling complex relationships among risk factors may substitute proper theories of disease causation and etiologic concepts. For example, Fatima et al. (9) found that most of the COVID-19 researchers used data-driven models rather than theory-driven methods. This approach may determine how disease and its causes are understood, potentially “explaining” variations in disease rates and influencing to certain ways the formulation of research agendas (50). Particularly in empirical spatial modeling such relations between model and explanation have been extensively discussed. Critical GIS has called for making very explicit how the system to study is described and how spatial scale, temporal scale, unit of analysis, spatial dependence, spatial interference and heterogeneity are considered (51, 52). Following this conceptual and theoretical dimension, methodological and methodical questions arise, such as how issues of modifiable areal unit problem or confounding variables are addressed (53). Open science principles advocate for transparency and reproducibility in research, which is particularly crucial in the context of public health emergencies. This paradigm emphasizes presenting empirical research openly, critically assessing findings, sharing data whenever possible and following FAIR (findable, accessible, interoperable, reusable) principles (54). While these aspects have all been identified in earlier studies already, it remains a key issue to be considered in empirical studies (55). Lastly, the instrumental dimension of deriving information for adequate health strategies adds an important perspective because the research studies are aimed to inform stakeholders and decision-makers.

Notwithstanding the growing awareness for the conceptual and methodological complexities involved, identifying and characterizing human health effects of the built environment is of particular interest to develop adequate health strategies. Specifically, with the recent COVID-19 pandemic, the built environment has been received considerable attention as an explaining factor for disease transmission and as a criterion for public health intervention (1). Spatial analysis of the patterns of COVID-19 and the analysis of associations with the local community is of high importance, particularly in the early stages of a pandemic (9). With the urban equity perspective (31), targeted measures are necessary for those with the most need or largest barriers besides the overall aim of providing healthy environments. This calls for spatial analyses of the built environment and health that not only assess spatial disparities but also consider the various vulnerabilities that are represented by particular groups. Revealing spatial inequities, however, comes along with an inherent ambivalence to consider. On the one hand, using locally collected data and adaptable tools to highlight and address local disparities allows for precise intervention and possibly facilitates meaningful change (33). On the other hand, statements regarding “infectious spaces” or “contagious neighborhoods” are politically performative, especially in the case of a pandemic and the accompanying public concern (50, 56). As sketched out above, such statements regarding the influence of the built environment on COVID-19 are methodologically demanding. Complexities regarding host-environment relations may easily be lost when translated to a general public and/or political decision-makers. This may lead to stigmatization (50, 56). It is specifically important to communicate findings clearly and to be wary of unsubstantiated claims and possible implications. With Zhong et al. (21), we constitute that there is still a knowledge gap in how to incorporate results on possible interlinkages between the built environment and health, into urban planning and policy to promote healthy communities and cities.

In summary, despite considerable research on the COVID-19 pandemic and the built environment, there remain significant gaps in our understanding of how these interactions are constituted (43, 57, 83). As part of a research project on the spatial relative risk for COVID-19 infections in the district of Berlin-Neukölln, we ourselves engaged with recent attempts to single out the spatial aspects of environmental influences in describing differences of COVID-19 incidence rates (1, 3, 35). Additionally, we are aware of prior guidelines concerning the management of health data (58) and explicit COVID-19 information (58, 59). We also acknowledge that discussions surrounding methodological challenges of COVID-19 studies have already been addressed elsewhere (60).

In this article, we now build on the current state of the debate and address in detail the intricacies of both explicit and implicit assumptions that stem from the utilization of data and methodologies from a critical perspective. More specifically, we aim to identify key caveats in empirical studies on spatial determinants of the built environment on urban health and to synthesize guiding principles for future studies. To do so, we analyze how the built environment in urban settings was studied in recent literature on COVID-19 using a set of criteria. The remaining article is structured as follows: in the second chapter, we first present our analysis approach and the respective criteria and then the results for recent studies on COVID-19 and the built environment in urban settings. In chapter 3, we systematize and discuss three typical entry points for inconsistencies in operationalizing spatial aspects in relation to COVID-19. Based on these “common errors” we found in the literature, we conclude our article with the formulation of guiding principles to increase viability of quantitative empirical research focusing on spatial determinants of the built environment on health.

## 2 Analysis of studies on the effect of the built environment on COVID-19

### 2.1 Literature analysis

To identify shortcomings and caveats in how spatial determinants of health are considered for modeling spatial determinants of the built environment on COVID-19, we undertook a semi-structured scoping review. Following the suggestions of Munn et al. (61) a scoping review specifically allows to examine how research is conducted on a certain topic, to identify key characteristics and to analyze knowledge gaps. We operationalize this scoping review in the following way: develop a systematic criteria approach for the analysis, following an explicit search scheme to identify relevant literature, present the data and results in a structured way, and increase reliability by having the criteria applied by three individual researchers.

We searched for articles from ISI Web of knowledge using the terms “COVID-19,” “built environment” and “urban” in the title, abstract, or keywords. We considered studies from various countries to capture a range of local settings, focusing exclusively on those that examined intra-urban settings and used quantitative modeling to identify spatial determinants of COVID-19 outcomes. Clinical trials were not considered for this review. In total, we included 23 peer-reviewed articles published in English language between January 2020 and January 2023 (see for an overview of Supplementary Table S1). Given the methodological difficulties in discerning spatial aspects in empirical research, as outlined above, our aim is to identify the underlying assumptions and caveats in the empirical studies and to formulate guiding principles. We do not try to evaluate the quality of the studies as such and the methods used, nor do we aim for a comprehensive review, but rather to assess the challenges and implications of their methodological approaches.

Hence, we iteratively developed the following set of evaluation criteria based on the identified challenges and the literature addressed in the introduction:

- Title and publication date: to capture the point in time of the study in this highly dynamic pandemic with the associated time pressure, increasing body of knowledge and temporal differences because of COVID-19 variants or measures.
- Study area: to capture different regional variations and location-specific characteristics of the COVID-19 pandemic.
- Aim of the study: to specify the objective of the study whether it is to identify the effect of the built environment on COVID-19, associations and variations in space and time or the prediction of risk.
- Process studied: to identify if a process is explained to describe the studied effect of the built environment on COVID-19, and if so, which one and with which underlying hypotheses.
- Methodological approach: to differentiate theory-driven studies that refer to existing theories and concepts from purely data-driven studies, or transitions between the two extremes.
- Outcome variable COVID-19: to differentiate various types of variables.
- Definition and indicators of “built environment” and confounding variables: to assess how the variable “built environment” is defined and empirically measured and which confounding variables are accounted for. Since this is the focus of this study, we report and classify the identified indicators in a more detailed form in Table 1. We classify them according to their type of meaning by relying on the four density types of McFarlane (39) and extend them by two more types, namely: built form (= the built physical and biophysical form), structures and interconnectivity (= structures that characterize the interconnectivity of places).
- Temporal scale: to characterize the temporal resolution of the study (e.g. weekly or daily) because of the high temporal dynamics of the pandemic.
- Spatial unit of analysis: to characterize the spatial resolution of the study (e.g. aggregated on the level of spatial units) because of the high spatial dynamics of the pandemic and the methodological challenges that are inherent.
- Spatial process: to identify whether underlying spatial processes, such as spatial dependency or autocorrelation are accounted for.
- Spatial inequity: to identify whether the spatial analysis of the built environment and health accounts for different vulnerabilities since this is a particularly important perspective on urban health (31).
- Data: to characterize the type of data that is being used and if data limitations are mentioned.
- FAIR Data principles: to assess if information according to FAIR (= findable, accessible, interoperable and reusable) principles are provided.
- Limitations: to identify if and what kind of limitations are referred to.

**Table 1:** List of indicators used to capture the built environment.

**Built environment**	**Dimension**	**Category**	**Indicators**
	Density	Density as numbers of people gathering at sites	• Land use mix (N) • Accessibility to CBD (km) • Accessibility to major activity centers (km) • Commercial area (%) • Commercial, industrial and educational buildings (N/km^2^) • Distance to parks, open space, playgrounds (km) • Office area (%) • Sidewalks of total area (%) • Parks of total area (%km^2^) • Number of clinics in the Tertiary Planning Unit (N) • Number of restaurants in the Tertiary Planning Unit (N) • Number of public markets in the Tertiary Planning Unit (N) • Employment and household entropy • Local facilities (N/km^2^) • Densities of shops (N/km^2^) • Densities of clinics (N/km^2^) • Densities of restaurants (N/km^2^) • Common POI data (catering, entertainment, hotel, medical, office, and culture) (N/km^2^) • DC student who lives and attends school in the same wards ratio (N)
		Density as numbers moving through space	• Distance to train station (km) • Distance to bus stop (km) • Intersection density (N/ha) • Road Networks: Nodes (N), Edges (N), Intersection Count (N), Streets per node... • Street length (km) • Number of entrances of Massive Transit Rail in the Tertiary Planning Unit (N) • Public transport (N/km^2^) • POI density around railway stations (N/km^2^) • Travel time by public transport (h) • Population flow (%) • Commute time to work (h) • Densities of bus stations (N/km^2^) • Modal share (%) • Road density (km/km^2^)
		Density as numbers of people living in an urban area	• Residential area (%) • Residential area with multi-story buildings (%) (higher than 3 floors) • Single-family residential buildings (N/km^2^) • One or two family units (%) • Building concentration (%) • Population density of built-up areas (N/km^2^) • Height to width ratio (%) • Frontal area density (%) • Floor area ratio (%) • Sky view factor (%)
		Density as numbers living in a house	• Living space (m^2^/N) • People per household (N) • Crowding ratio (%) • Rooms per person (N) • Household composition (single, family with children….)
	Built form	The built physical and biophysical form	• Park area (%) • Tree cover (%) • Apartment (yes/no) • Dwelling size (m^2^) • Building geometry (Building height, Building density, Sky view factor) • Green space (%) • Normalized difference vegetation index (NDVI) • Retirement homes (N/km^2^) • Average age of construction • Housing age • Housing size • Housing energy efficiency • Greenspace ratio • Type of housing (public, temporary…) • Tenure (ownership, tenant)
	Structures	Structures and interconnectivity	• Regional auto centrality index • Regional-international connectivity, size, density, form • Betweenness centrality • Median value of the shortest distance between the entrance of MTR and the residential buildings in the Tertiary Planning Unit (km) • Median value of the shortest distance between the public market(s) and the residential buildings in the Tertiary Planning Unit (km) • Median value of the shortest distance between the restaurants and the residential buildings in the Tertiary Planning Unit (km) • Median value of the shortest distance between the clinics and the residential buildings in the Tertiary Planning Unit (km)

The 23 studies identified according to the search pattern were evaluated according to these criteria. This differentiation is presented in the following chapter. Based on these findings, we identified three key challenges of empirical analysis of built environment and COVID-19 and finally derive recommendations for future studies.

### 2.2 Results of the literature analysis

In the following, the results of the literature corpus analysis of the 23 papers are briefly summarized for each criterion. The detailed table is available on request from the authors:

**Title and publication date:** the subset we included ranged from July 2020 to January 2023 (2020:1, 2021:14, 2022:2, 2023:6).
**Study area:** study areas span across the globe with a regional concentration in the US, Europe, and Asia. The following countries were included: Australia, China and Hong Kong, England, Germany, Greece, Italy, USA. All the study areas are primarily located in urban settings.
**Aim of the study:** all studies focused on the relationship between COVID-19 and built-environment and socio-economic variables, yet with different foci. The thematic focus was mainly either on the effect of the built-environment, i.e. land use, housing conditions, building geometry, urban density and/or connectivity, on COVID-19 (2, 62, 63, 88) or on behavioral responses to social-distancing policies (46, 83). From a methodological perspective, the relationship of built environment effects on COVID-19 over time was studied (35) or the local spatial variations (1, 2) or a combination of both. Almost all studies aimed for exploring the associations (64, 65) and two studies explicitly aimed for explaining or predicting the risk of a COVID-19 infection (40, 62).
**Process:** in line with the aim of the studies, the processes addressed, even though only rarely explicitly mentioned in the articles, are mainly related to the transmission of COVID-19. The underlying assumption of all studies, although not always clearly stated (57, 65), is that COVID-19 transmits between people through close contact. Two articles state that high housing densities (measured as the place of residence) are assumed to reflect high transmission rates [following findings from (43, 57)] and warn that dense urban environments promote more interactions and greater proximity among people thereby increasing the risk of spreading. Other hypotheses suggest that neighborhoods with highly diversified land use and connectivity and transportation infrastructure may foster greater congregation and interactions and therefore quickly spread COVID-19 (66). A few studies discuss the association between greenspaces and COVID-19 infections (2, 35, 40, 65, 67). On the one hand, greenspaces may improve immunity through physical activity, while on the other hand, its use during the pandemic may promote close contacts and increase the risk of infections (40). Only two studies discuss the issue of the actual place of infection, e.g. the workplace (2, 68), but none have systematically analyzed these locations due to a lack of available data. All processes that are addressed can be categorized as indirect pathways of the built environment.
**Methodological approach:** almost all (19 from 23 studies) studies are primarily data-driven, focusing on empirical data collection and analysis while placing minimal emphasis on existing theories or concepts (46, 57, 64, 67). In contrast, no study employed a fully theory-driven approach, which we define as one where theories and concepts are explicitly integrated throughout the research process, with references included in both, the introduction and discussion. At the same time no study relied solely on data analysis without referring to existing theoretical state of the art either. Five studies combined data- and theory-driven approaches, introducing a theoretical framework as a guide but not consistently addressing it in the discussion (35, 63, 69, 70, 85).
**Outcome variable:** the COVID-19 outcome variable used in the studies varied, with almost all studies (18 of 23 studies) focusing on incidences (positively tested cases per inhabitants) and 5 examining fatalities or hospitalization (43, 46, 65, 70, 85). In two out of three studies, the data was aggregated based on point level address data (registered residential address) (40, 67).
**Built environment and confounding variables:** interestingly, some articles address the built environment in their title or aims of the study but lack to include a clear definition of the term (46, 71). The analysis of how indicators were selected to measure the effect of the built environment, as well as the extent and quality of available information, revealed that the built environment is predominantly described as “human-made spaces.” However, the indicators used are very diverse. In all studies (40, 57, 62), built environment is captured and described by multiple indicators (see Table 1).

We classify these indicators according to their type of meaning by relying on the four density types of McFarlane (39) and extend them by two more types, namely: built form (= the built physical and biophysical form), structures and interconnectivity (= structures that characterize the interconnectivity of places). Each indicator was attributed to the one best-fitting category in the Table 1. Indicators that were named in different articles, were only reported once here. The number of considered indicators varies significantly [e.g. up to 125 variables in (67)]. All studies accounted for other influencing factors beyond built environment, though the transparency of this consideration varied. For example, one study explicitly referred to control variables such as median age of the population (40). The following indicators were used frequently: total population (N), population density (N/km^2^), age, ethnicity, marital status, spoken language, educational attainment, economic activity status, place of work, monthly income, weekly working hours, household income.

**Temporal scale:** we examined the temporal scale by reviewing the time periods covered in the studies and determined whether they divided the study period into different phases of the pandemic. In our set of literature more than half of the studies focused on the early beginnings of the pandemic during the first phases (from March 2020 onwards) but with very different time frames, e.g. a couple of weeks, multiple months, more than a year. Only three studies divided the study period into different phases (mostly characterized and defined by different measures) (3, 35, 84). Several temporal challenges were addressed, including reporting biases (e.g. variations in daily or weekly reporting), while others attempted to capture dynamics in the COVID-19 data but were limited by missing data on the dynamics of, for example, densities or contacts. As a result, evaluating the effects of measures remained challenging.
**Spatial unit of analysis:** we found that all studies used aggregated units for analysis, which varied widely. These units ranged from administrative boundaries [e.g. ZIP-Codes (57) or city boundaries], to statistical units [Tertiary Planning Unit in Hong Kong (64); census tracts in the US (70)], to specifically developed neighborhoods for planning [neighborhoods in Bochum (2), planning units in Berlin (35), Wards in Washington (65)] to regular grids (500 × 500 m grids) (62). The selection of the spatial unit of analysis most often depends on the availability of contextual data since COVID-19 data is aggregated to the targeted unit of analysis. However, discussions about the spatial aggregation and the interpretation of results at different spatial scales are frequently lacking.
**Spatial processes:** when examining the approaches taken to address spatial processes, we paid close attention to the mapping and to the assumptions made regarding spatial heterogeneity, dependency, or homogeneity. Almost all studies assume spatial dependency and heterogeneity and address this to different degrees in the empirical analysis. About half of the studies tries to incorporate spatial dependencies in their modeling approaches (2, 40), however, there are also multiple studies that use global models without looking specifically at local heterogeneities (62, 64). Spatial autocorrelation is explored and respective indices (e.g. Moran's I and LISA) were calculated by four studies (1–3, 40), only one study explicitly focused on urban-rural gradients with varying degrees of built environment (68).
**Spatial Inequity:** spatial inequity, captured by spatial disparities in health risk for different social groups, is addressed in more than half of the articles (68, 87). In two out of three studies, it is referred to disparities in race, ethnic minorities and social status (65, 70, 84). What is being stressed by many studies is that understanding social distancing and behavioral change in neighborhoods can inform more effective public health policy, though the impact of these interventions at the neighborhood level remains largely unexplored despite awareness of disparities in vulnerable communities (3, 46).
**Data:** to capture these aspects, the indicators depicted in Table 1 predominantly used available statistical data on exposure (57, 68) and the built environment (e.g. urban structure types). In two cases, remote sensing data (2, 62) was analyzed and one study used anonymized smartphone geolocations for exposure analysis (46). Mostly, the data was preprocessed and used as an index [NDVI Normalized Difference Vegetation Index (62), Shannon-Index to capture diversity (40)], or as indicators (see Table 1 for built environment). Data limitations were identified in more than half of the studies (2, 40, 46, 83), such as the need for additional datasets and the fact that explanatory variables were often only available and used at a single point in time.
**FAIR data principles:** only one study provided information according to FAIR (= findable, accessible, interoperable and reusable) principles (2). Another study offered FAIR data to a very limited degree by offering a tool for further data exploration (3). Two out of three studies referred to existing data to other sources (2, 40, 46). No study provided all datasets for free accessibility. Very often data privacy issues were referred to as a reason for difficulties in sharing the data (1). Documentation of the data varied to a large degree, and we assume that only few studies would be reproducible with the provided information.
**Limitations:** limitations of the undertaken modeling studies were mentioned in three out of four studies, but not in all articles (57, 62, 65). Identified limitations range from the categorized temporal resolution of COVID-19 data, where the defined phases may not accurately match the real transition from one phase to another. The fact that the place of residence is not necessarily the place of transmission was frequently mentioned. Additionally, a need for the inclusion of more variables to account for the complexity of the urban system or the need to refine and detail the used variables (e.g. in terms of different land use types or the vegetation-covered are in terms of quality and accessibility or changes in population numbers throughout the pandemic) was discussed (2). The drawback of ecological analyses that do not control for other potential risk factors (e.g., the usage of public transport or occupation in high-risk jobs) was highlighted several times as well as missing information on individual behavior (e.g. wearing masks) and the challenges associated with the unit of analysis [e.g. census tracks (64)].

## 3 Identified challenges in empirical studies on the effect of the built environment on COVID-19

Based on the above-presented findings on the characteristics of how built environment is addressed in current COVID-19 literature, we identify the following three key caveats of empirical modeling: (1) The critical reflection of underlying assumptions, (2) Representation of the urban health system and processes via indicators in an empirical study, and (3) Spatial and temporal dynamics of the processes modeled. We undermine these with references from the body of literature studied and discuss these in the light of additional studies.

### 3.1 Critical reflection of underlying assumptions

Our most important finding is that the underlying assumptions regarding the representation of the urban health system and its complexity, the addressed process and particularly the built environment are often not considered and/or presented in a consistent and transparent manner in most articles (46, 57, 65, 71). Most importantly, only few studies critically reflect on the inherent challenges of their individual approaches and empirical analyses (1, 2, 71). This can result in the prolongation of hypothesized spatial effects without actual empirical proofs. The narrative of dense urban areas as a historical hotspot of high health risks or of an inherent contagiousness of built environments often serve as a foundation for empirical analysis (57, 67). We see a tendency to repeat this narrative in the conclusions, even when the findings are not substantial enough to warrant those claims. As Florida (72) states, “many inspecting the spread trend of this novel virus rushed to blame density,” making it one of the most controversial and influencing factors that stood out puzzling many and delivered questions as to whether urban density and healthy cities can be related in regard to COVID-19. The built environment, with its various associated meanings, provides a telling example to distinguish two different perspectives here. A space-based perspective aims to discern a direct ‘contagiousness' of certain types of built environment (e.g. relationship between high-density buildings and case numbers). A place-based perspective in contrast tries to understand the effect of perception. A certain imagination of pathogenic qualities of certain types of built environment already may change peoples' behavior and such may have a health effect (e.g. high-density buildings perceived as risky could prompt behavioral changes, such as avoiding shared spaces). Most of the analyzed studies adopt a predominantly data-driven methodological approach, with limited integration of theories and concepts throughout the research process which has also been identified in an earlier review of COVID-19 studies (9). Whether a space-based or place-based hypothesis is underlying remains implicit for example. Finally, the underlying aim of a modeling approach varies from an exploratory analysis that identifies associations between different variables to a predictive or explanatory approach. A careful distinction of the—even though well-known—difference between statistical association and causal inference is necessary and implicit shifts in the line of argumentation need to be prevented.

### 3.2 Representation of the urban health system and processes via indicators in an empirical study

While there is a large body of literature on theories investigating urban health and the role of the built environment including reconceptualization, an examination of political governance scales, or a postcolonial critique of urban theory, we can confirm and extend with our findings the argument by Hu et al. (65) that there “has been a deficiency in directly connecting these theoretical models to public health and more categorically to social determinants of health.” This gap is particularly evident for spatial determinants, such as the built environment.

Faced with the complex category of “built environment,” the studies must discern certain aspects of epidemiological relevance that guide the operationalization and especially the selection of data. Researchers are therefore forced to capture fuzzy concepts related to the effects of space by translating them into distinct variables and datasets. The large variation in how the built environment is defined and the types and number of built environment variables used in the 23 differentiated studies reflects this fuzziness (64, 65, 67). Typically, “density” (including crowding), “structure and interconnectivity” and “built form” are identified as epidemiological relevant aspects of the built environment and characterized by indicators (40, 83). A common challenge is to keep those aspects consistently defined throughout the study or when referring to existing research. The term “built environment” holds e.g. very different qualities of space, i.e. space as relational concept, space as container, space a place. Only rarely, the studies discuss the difference between the meaning that is given to space and the meaning it has, frequently addressed as spatial trap (73). The same is true for density, which conveys, for example, different meanings as also systematically discussed by McFarlane (39). Different situations of spatial interaction in the cities are termed “dense” regarding COVID-19, each with a different process of contagion and infection assumed (42). If “density” is captured by the number of people living in an urban area, this variable is mostly a proxy for “cityness” (high number of services, apartment-type housing, pedestrian interactions) (40, 57). In this case, a significant density–infection relation shows a higher risk of city-users to contract COVID-19. Only in this case is the assumed effect directly related to the built environment. In contrast, if density is used to denote the number of people living in a house, it is much more a socio-demographic proxy. Localizing the exact moment of viral transmission is rarely possible and thus the home cannot reliably be established as the site of transmission. A significant effect here does not allow to make claims about the built environment then. It hints toward the higher risk of a certain socio-demographic group (those who only can afford small individual living space) to contract COVID-19. A third meaning denotes density as the numbers moving through space, including transport systems, streets and the in-between spaces of city-center shopping, and so on (70, 83). A significant effect here would allow to assume daily interactions, in trains, at workplaces, in shops and at schools as the drivers of infection.

Capturing important variables in a modeling approach is a fundamental challenge. However, only a few studies account for confounding variables like age or socioeconomic status (35, 40, 43, 64). Moreover, only few studies describe and discuss in detail the reasoning for the choice and operationalization of the indicators they use (63, 83). We refer here to Rothenberg et al. (33) and Pineo et al. (29) who provide a good overview of urban health indicators (not linked to COVID-19) and particularly call for locally adapted flexibility. We echo Rothenberg et al. (33) that “perhaps the real power of indicators and indices is to demonstrate disparity on the local level—a place where significant change may be possible. Locally collected data and simple, flexible tools for amalgamation, rather than fixed packages, may be a fruitful approach to understanding health disparity.”

### 3.3 Spatial and temporal dynamics of the processes modeled

The associations between COVID-19 and the built environment, as with many other health outcomes, exhibit high spatial and temporal dynamics with significant spatial dependency. However, this is only in some articles explicitly considered and discussed (40). While variations in time and space are obvious from a process-based understanding of COVID-19 infections, many studies still use space and/or time as containers. For example, some studies applied global correlation approaches without addressing local dependencies (62, 64), while others did not differentiate between the various phases of the pandemic (2, 57). Similar to Fatima et al. (9), who identified data quality as the main limitation of any spatial analysis that determined the use of spatial techniques and methods, we also recognize the need for the availability of more detailed data to derive evidence-based information.

In regard to space, this includes the very fundamental reduction of complexity by focusing on specific points in space, such as homes or workplaces, and neglecting the daily mobility patterns. Therefore, more research is needed to examine daily mobility and activity spaces in detail to better understand transmission dynamics. Furthermore, the chosen unit of analysis (and the corresponding modifiable area unit problem), the spatial (and multi-) scale effect and the level of aggregation, which can lead to issues like ecological fallacy need to be accounted for. Additionally, it encompasses the challenge of balancing local specifics with the need for comparability and transferability or general applicability of processes to other regions. For example, since many studies adopt a locally specific administrative approach to delineate the spatial features, the spatial units often differ significantly in terms of population size and areal extent across studies [e.g. planning units in Berlin (35) vs. wards in Washington (65)]. Also, some studies discuss their findings that deviate from other studies, attributing these differences to the very unique type of built environment that they observe in their case studies (62). Further challenges in urban areas involve the definition of the neighborhood and being aware of the problem of the modifiable unit in that statistical, census, transport, or planning units have certainly not been delineated with epidemiological questions in mind.

In addition to the spatial dimension, the temporal dimension is key for COVID-19 studies, as incidences have been especially influenced by the changing regimes of epidemiological interventions both across places and time periods (40). This is often not considered in regression analyses. Instead, the changing context is assumed to be stable or is not accounted for at all. For example, dominating transmission processes varied over time during the pandemic with differences in measures such as school closures. Reasons for this often include challenges related to reporting bias (e.g. variations in daily or weekly reporting), with dynamics typically addressed only within COVID-19 itself but not in relation to changes in density, contact patterns or other dynamics due to missing data. Consequently, it becomes very difficult to evaluate or estimate the effects of measures. Additionally, it is important to recognize that different phases of the pandemic in various countries were shaped by distinct factors, with the virus's spread driven by complex causal chains that primarily impacted specific groups (e.g., socioeconomic or age groups) at different times (3, 74, 75). These shifting patterns of impact pose interpretative challenges, complicating both the results and methodologies of studies and making it difficult to draw consistent conclusions across diverse contexts.

In addition to these three key challenges, we now discuss additional observations based on our findings. First, the studies were published rapidly due to the urgency of the emerging disease and associated challenges (43, 71). For an exploration of a newly developing health situation this is of utmost importance, but it should be good scientific practice to critically reflect on these circumstances within the publications. We also identified a few papers with overall lower study quality, which may be attributed to the urgency and rapid pace of the pandemic and the need for quick knowledge generation. One suggestion for future publishing in such cases might be to focus more on pre-prints and online discussions/review and use the possibility to comment and revise as soon as further knowledge is available. Second, we found that study areas span across the globe with a regional concentration in the USA, Europe and Asia, which is largely due to the analysis of only peer-reviewed scientific articles in English language. We assume that the location of the empirical study and the authors' backgrounds might bias, for example, the selection of questions, data analyzed, and urban processes studied. For future studies, comparative approaches might offer new insights and can account for different regional processes. Third, the aims of the study often seem driven by data availability rather than addressing the processes of interest. For example, the COVID-19 data collected by the public health departments was not explicitly designed for empirical health research. Moreover, it seems of utmost importance to develop and make available new datasets that capture additional facets of density and built environment, such as crowding or workplace density. Particularly important is the availability of large-scale health data such as COVID-19 case data. These would open new possibilities for research. The same holds true for the availability of data that reflects dynamics in population or mobility numbers. A critical reflection of new sources (anonymized individual level data, activity patterns from smartphone data etc.) in the light of ethics and data privacy should take place as well as a consideration of new developments in terms of data infrastructure and management (58, 59).

The aim of this study was not to comprehensively summarize the current state-of-the-art but to distill common challenges and, most importantly, formulate guiding principles for future studies. Our exemplary corpus of 23 studies allowed to present new insights into common challenges of modeling spatial determinants of health for the example of the built environment and the association with COVID-19. We are aware of some limitations of this approach. First, we only considered articles published in English language and accessible via the ISI-Web of knowledge. We also focused on the early body of literature to capture the direct response in research to this newly emerging public health challenge. This might have led to a particular set of quickly published studies in our analysis and we did not account for quality of the studies explicitly but assumed that the publication in peer-reviewed journals is a minimum quality standard. Moreover, in addition to the search term “built environment,” one could have included additional terms such as “architecture” or “built structure,” however, the objective of this article was not to conduct a comprehensive systematic review but it's aim was to use “built environment” as a focal example since previous studies have identified it as a key determinant of health. Finally, while we did not consider all research articles addressing COVID-19 and the built environment, we believe that the set of 23 studies selected for our scoping review provided a good basis for deriving the following guiding principles.

## 4 Summary and guiding principles

**Figure 1:**
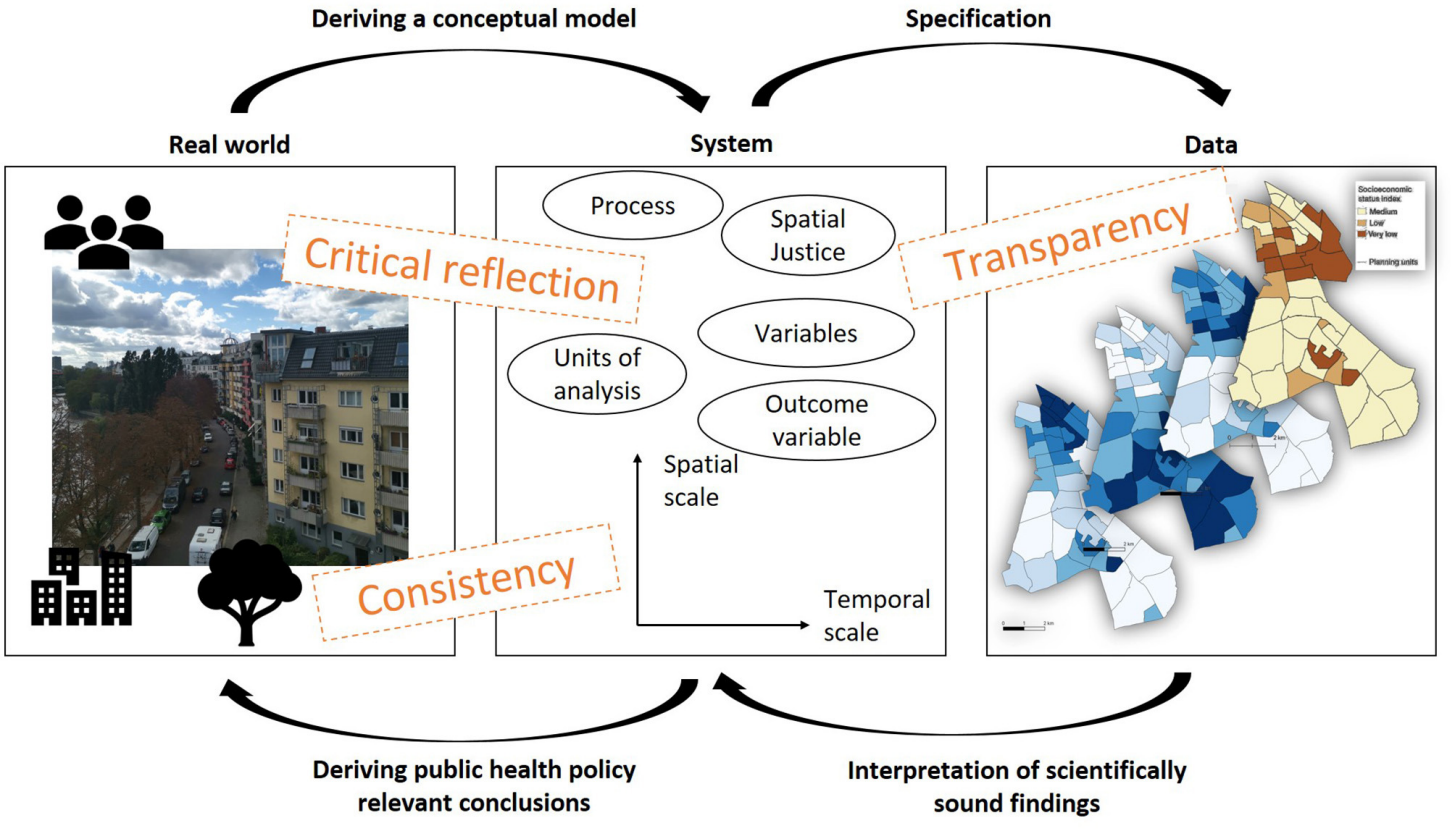
Modeling urban health challenges via a system approach for public health policy relevant conclusions.

The analysis revealed important reflections on how studies approach modeling spatial determinants of health, in this case relationships between the built environment and COVID-19 transmission. We now conclude our text with the following guiding principles and questions for future research along the values of transparency, consistency and critical reflection:

1) Authors need to reflect and critically discuss the underlying assumptions of the system in their study.
In empirical studies, it is essential to reflect on the relevance of underlying theories and established state-of-the-art knowledge, considering existing assumptions and case-study specific contributions to the overall body of knowledge (see Figure 1). Pure data-driven case-study-specific analyses should be avoided, or if necessary, clearly specified as such without deriving any causal conclusions or policy recommendations. The aim of a specific modeling experiment should be clearly defined as well as the process it addresses (e.g. the transmission of COVID-19).
  - Is the aim of the modeling experiment clearly defined?
  - Is the underlying process defined?
  - Are existing theories and concepts referred to in the specification of the conceptual model (e.g. the identification of the outcome and explanatory variables and the indicator development)?
  - Are policy-relevant recommendations based on empirically sound information?

2) Aspects of spatio-temporal effects on health need to be clearly and transparently defined and kept consistent throughout a study
  The spatial dimension in empirical health studies should be considered explicitly. This includes the definition of the unit of analysis, the choice of the spatial scale of analysis, spatial heterogeneity and spatial dependency in the data. They need to be described and, as far as possible, accounted for in the empirical experiments and critically reflected. Moreover, spatial effects captured via indicators should be addressed consistently without changes in meaning (see the example of density above) throughout a study. Similarly, temporal dynamics need to be included and maintained consistently.
  • What are spatial characteristics of the process studied and the used data?
  • What are the temporal dynamics of the process?
  • Which spatial effect is represented in an indicator and is it consistent throughout the study?
  • How are spatial effects visualized in maps and are they critically reflected?

3) A critical reflection (a-priori) is needed to determine if a process can be studied with a particular study design or if other methods would be more adequate
  - Is the study design adequate for answering the research question? For example, are data on actual activity patterns needed in contrast to the reduction to places of residences?
  - Are contingencies of space and inherent dynamics considered? For example, the double quality of space produced by and producing social relations?
  - Are statistical associations clearly separated from causal inferences?

4) Providing new datasets and best practice examples for datasets that were not originally collected for research analysis but provide important insights
  - Which datasets from public or private stakeholders in the public health sector are being assessed and can these datasets be made available for research?
  - Which datasets and scripts from research studies can be shared?
  - How can “failed experiments” or studies with contradictory results be shared?
  - How can FAIR data principles be supported?

While these overarching recommendations and guiding questions may be key, however, we see that in case of a new pandemic, the specific settings will potentially complicate their application. The immanent time pressure, the little prior theoretical knowledge, the high dynamics of the pandemic in space and time all complicate their application. It is notwithstanding important to adhere to careful and reflective approach in order to avoid misleading or stigmatizing conclusions as the knowledge generation in such a situation may be especially policy-relevant. We believe that while focusing on COVID-19, our findings may also stimulate more general discussions on how to approach empirical urban health analyses, particularly as associations between the built environment and other health outcomes have been intensively studied [e.g. heat stress (76), vector/waterborne diseases (77), mental illness (78) or chronic diseases such as cardiovascular, diabetes, cancer or cognitive decline (79–81)].

Finally, our findings of the studies on COVID-19 highlight that the small-scale intra-urban empirical analyses of COVID-19 calls for new discussions and guidelines not only in regard to the above illustrated conceptual and methodological caveats but also in terms of data management [FAIR data, e.g. (58, 59)], legal (data privacy), ethical (stigmatization) and political communication (what to do with the findings) aspects. Only critical, sound and transparent research can be a basis for the necessary inclusion of health aspects in urban planning and policy to move forward to more resilient, sustainable cities and that not only risks but the built environment itself as a possibility to develop healthy environments should be considered (82).

## Data Availability

The original contributions presented in the study are included in the article/Supplementary material, further inquiries can be directed to the corresponding author.
