# Supplementary material for: Pathogenic built environment? Reflections on modeling spatial determinants of health in urban settings considering the example of COVID-19 studies
Source: Front Public Health. 2025 Mar 17;13:1502897. doi: 10.3389/fpubh.2025.1502897 (PMC11955651; doi:10.3389/fpubh.2025.1502897)
Supplement: Supplementary file 1 [file Table_1.docx]

S Table 1: Study sample

| **Author(s)** | **Title** | **Year of publication** | **Study Area** |
| --- | --- | --- | --- |
| AbouKorin, S. A. A., Han, H., and Mahran, M. G. N. | Role of urban planning characteristics in forming pandemic resilient cities – Case study of Covid-19 impacts on European cities within England, Germany and Italy | 2021 | 27 cities in Europe (England, Germany, Itöay |
| Frank, L. D. and Wali, B. | Treating two pandemics for the price of one: Chronic and infectious disease impacts of the built and natural environment | 2021 | USA (Counties) |
| Gaisie, E., Oppong-Yeboah, N. Y., and Cobbinah, P. B. | Geographies of infections: built environment and COVID-19 pandemic in metropolitan Melbourne | 2022 | Melbourne, Australia |
| Hamidi, S., Ewing, R., and Sabouri, S. | Longitudinal analyses of the relationship between development density and the COVID-19 morbidity and mortality rates: Early evidence from 1,165 metropolitan counties in the United States | 2020 | USA (Counties) |
| Hong, B., Bonczak, B. J., Gupta, A., Thorpe, L. E., and Kontokosta, C. E | Exposure density and neighborhood disparities in COVID-19 infection risk | 2021 | New York, USA |
| Hu, M., Roberts, J. D., Azevedo, G. P., and Milner, D. | The role of built and social environmental factors in Covid-19 transmission: A look at America’s capital city | 2021 | Washington DC, USA |
| Kashem, S. B., Baker, D. M., González, S. R., and Lee, C. A. | Exploring the nexus between social vulnerability, built environment, and the prevalence of COVID-19: A case study of Chicago | 2021 | Chicago, USA |
| Kwok, C. Y. T., Wong, M. S., Chan, K. L., Kwan, M.-P., Nichol, J. E., Liu, C. H., Wong, J. Y. H., Wai, A. K. C., Chan, L. W. C., Xu, Y., Li, H., Huang, J., and Kan, Z. | Spatial analysis of the impact of urban geometry and socio-demographic characteristics on COVID-19, a study in Hong Kong | 2021 | Hong Kong, China |
| Lambio, C., Schmitz, T., Elson, R., Butler, J., Roth, A., Feller, S., Savaskan, N., and Lakes, T. | Exploring the Spatial Relative Risk of COVID-19 in Berlin-Neukölln | 2023 | Berlin, Germany |
| Lee, W., Kim, H., Choi, H. M., Heo, S., Fong, K. C., Yang, J., Park, C., Kim, H., and Bell, M. L. | Urban environments and COVID-19 in three Eastern states of the United States | 2021 | New York, New Jersey, Connecticut, USA |
| Li, S., Ma, S., and Zhang, J. | Association of built environment attributes with the spread of COVID-19 at its initial stage in China | 2021 | 368 Chinese cities |
| Liu, C., Liu, Z., and Guan, C. | The impacts of the built environment on the incidence rate of COVID-19: A case study of King County, Washington | 2021 | King County Washington, USA |
| Mouratidis, K. and Yiannakou, A. | COVID-19 and urban planning: Built environment, health, and well-being in Greek cities before and during the pandemic | 2022 | Athens and Thessaloniki, Greece |
| Niu, Q., Wu, W., Shen, J., Huang, J., and Zhou, Q. | Relationship between Built Environment and COVID-19 Dispersal Based on Age Stratification: A Case Study of Wuhan | 2021 | Wuhan, China |
| Schmiege, D., Haselhoff, T., Ahmed, S., Anastasiou, O. E., and Moebus, S. | Associations Between Built Environment Factors and SARS-CoV-2 Infections at the Neighbourhood Level in a Metropolitan Area in Germany | 2023 | Essen, Germany |
| Schmitz, T., Lakes, T., Manafa, G., Lambio, C., Butler, J., Roth, A., and Savaskan, N. | Exploration of the COVID-19 pandemic at the neighborhood level in an intra-urban setting | 2023 | Berlin, Germany |
| Tepe, E. | The impact of built and socio-economic environment factors on Covid-19 transmission at the ZIP-code level in Florida | 2023 | Florida, USA |
| Tribby, C. P. and Hartmann, C. | COVID-19 Cases and the Built Environment: Initial Evidence from New York City | 2021 | New York, USA |
| Wali, B. and Frank, L. D. | Neighborhood-level COVID-19 hospitalizations and mortality relationships with built environment, active and sedentary travel | 2021 | King County Washington, USA |
| Xu, Y., Guo, C., Yang, J., Yuan, Z., and Ho, H. C. | Modelling Impact of High-Rise, High-Density Built Environment on COVID-19 Risks: Empirical Results from a Case Study of Two Chinese Cities | 2023 | Hong Kong and Shanghai, China |
| Yip, T. L., Huang, Y., and Liang, C. | Built environment and the metropolitan pandemic: Analysis of the COVID-19 spread in Hong Kong | 2021 | Hong Kong, China |
| Zhuang, S., Wolf, K., Schmitz, T., Roth, A., Sun, Y., Savaskan, N., and Lakes, T. | Neighborhood-level inequalities and influencing factors of COVID-19 incidence in Berlin based on Bayesian spatial modelling | 2024 | Berlin, Germany |
